# Supplementary material for: Reversal of nanomagnets by propagating magnons in ferrimagnetic yttrium iron garnet enabling nonvolatile magnon memory
Source: Nat Commun. 2023 Mar 29;14:1490. doi: 10.1038/s41467-023-37078-8 (PMC10060572; doi:10.1038/s41467-023-37078-8)
Supplement: Supplementary file 1 — Supplementary Information [file 41467_2023_37078_MOESM1_ESM.pdf]

Reversal of nanomagnets by propagating magnons in ferrimagnetic yttrium iron garnet enabling nonvolatile magnon memory

Korbinian Baumgaertl[1] and Dirk Grundler[1,2]

[1]*Laboratory of Nanoscale Magnetic Materials and Magnonics, Institute of Materials (IMX), École Polytechnique Fédérale de Lausanne (EPFL), 1015 Lausanne, Switzerland*

[2]*Institute of Electrical and Micro Engineering (IEM), École Polytechnique Fédérale de Lausanne (EPFL), 1015 Lausanne, Switzerland*

(Dated: February 9, 2023)

SUPPLEMENTARY INFORMATION

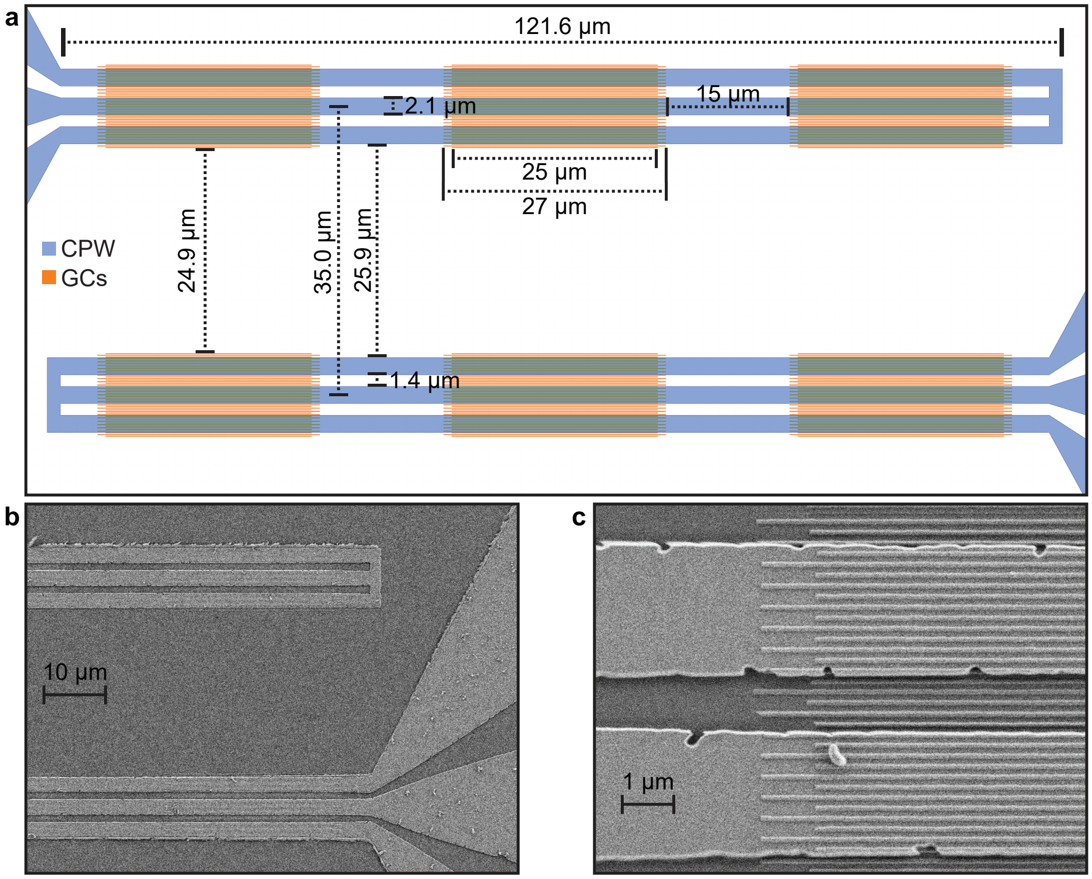

Supplementary Fig. 1. | **Layout and scanning electron microscopy images of the sample. a** Sample layout. Two coplanar waveguides (CPWs) (blue color) with a total length of 121.6 µm are separated by a center-to-center distance of 35 µm. Below each CPW three 25 µm long Py grating couplers (GCs) are arranged. Every second stripe of the GCs is prolonged on both sides by 1 µm and then its length amounted to 27 μm. The edge-to-edge separation of neighboring GCs underneath one and the same CPW amounts to 15 µm to avoid dipolar interaction. **b** Scanning electron microscopy image showing both CPWs with GCs. **c** Zoom in on the end of one of the GCs.

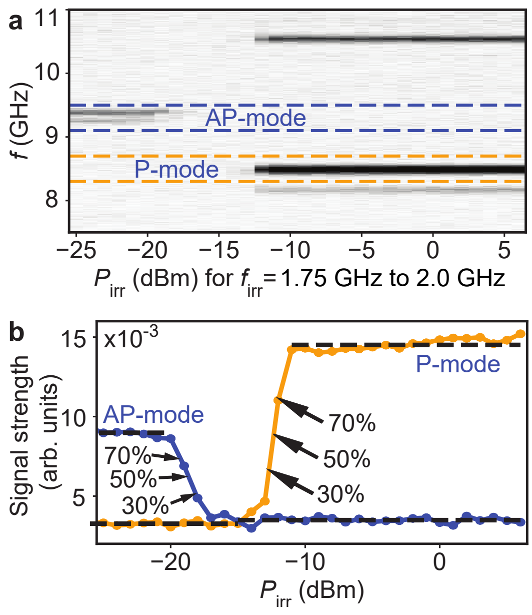

Supplementary Fig. 2. | **Evaluation of critical power levels P_{C1} and P_{C2}. a** Stripes were magnetized along $-y-$direction and a small power of $P_{\mathrm{sens}} = -25$ dBm was used to measure Mag(S21). Staying at $\mu_0 H_B = +14$ mT a microwave signal of power P_{irr} was applied in 0.25 GHz wide windows exemplary plotted for $f_{\mathrm{irr}} = 1.5$ GHz to 1.75 GHz and increased in a stepwise manner. After each increment of P_{irr}, Mag(S21) was measured with the small power P_{sens} between 7.5 GHz to 12.5 GHz. A transition from the AP-mode (enclosed by blue dashed lines) to the P-mode (enclosed by orange dashed lines) as function of P_{irr} was observed. **b** We quantify critical power levels P_{C1} and P_{C2} by evaluating the 50 % signal strength transitions of (AP-) (blue) and P-mode (orange), respectively. Here, AP (P) stands for antiparallel (parallel) configuration of Py and YIG magnetization vectors. We argue that the decay of SW amplitudes below CPW1 and CPW2 and the inhomogeneous stripe reversal in the grating couplers observed by MFM explain why AP- and P-mode transitions appear gradual and are not sharp step functions.

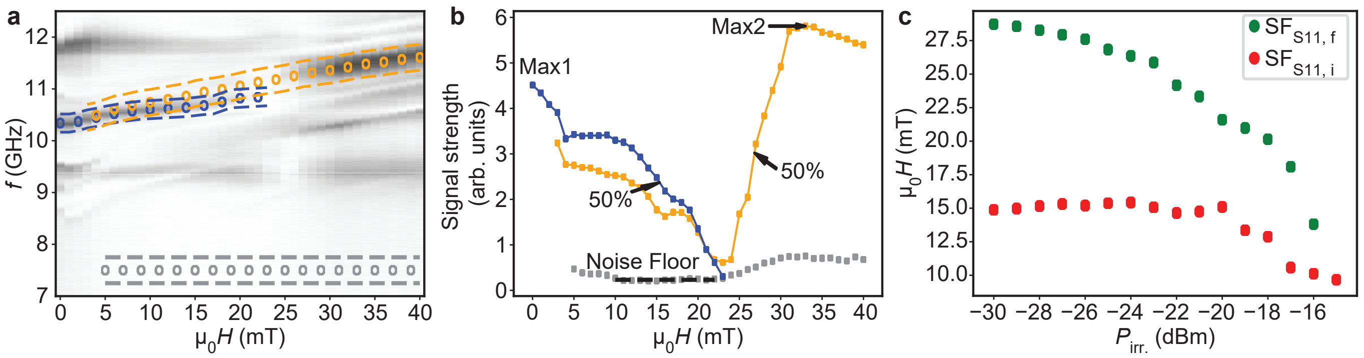

Supplementary Fig. 3. | **Power-dependent switching field distribution of Py nanostripes underneath CPW1. a** Exemplary Mag($S11$) for $P_{\mathrm{irr}} = -25$ dBm. We track a characteristic mode of the AP-configuration (blue dots) and the P-configuration (orange dots). **b** We quantify the switching field $SF_{\mathrm{S11,i}}$ ($SF_{\mathrm{S11,f}}$) by evaluating the decay (rise) of the signal strength to 50 % of its maximum value with respect to the noise floor (estimated from the region between the gray dashed lines in **a**). **c** $SF_{\mathrm{S11,f}}$ diminished more significantly than $SF_{\mathrm{S11,i}}$ with P_{irr}. Above $P_{\mathrm{irr}} \geq -15$ dBm, $SF_{\mathrm{S11,i}}$ and $SF_{\mathrm{S11,f}}$ were separated by less than 1 mT and automatic evaluation with the previously introduced methodology (cf. **a, b**) was not performed.

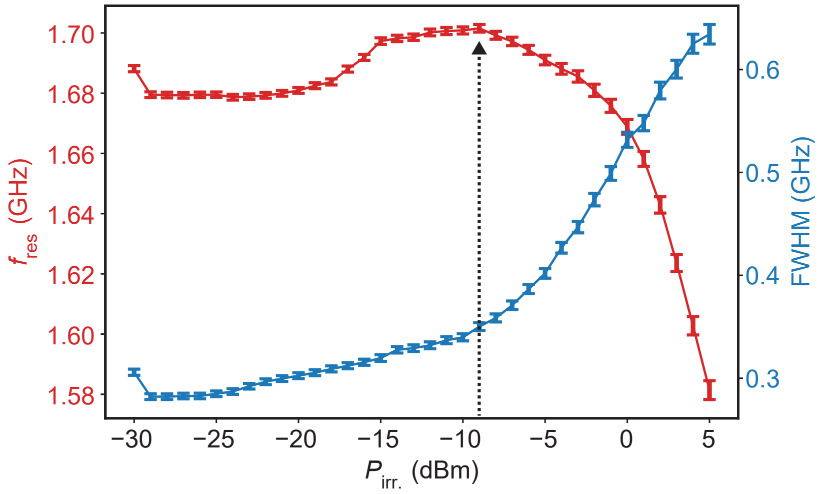

Supplementary Fig. 4. | **Onset power level of nonlinear spin-wave regime.** Power dependence of resonance frequency f_{res} (red color) and full width half maximum (FWHM) of the k_1 mode resonance in Mag($S11$) extracted by fitting with a Lorentzian function at $\mu_0 H = -10$ mT. Above $P_{irr} = -9$ dBm (indicated by dashed arrow) f_{res} starts to decrease significantly with increasing power. Around the same P_{irr} the FWHM starts to increase significantly. These two observations are attributed to the onset of the nonlinear regime of SW excitation.

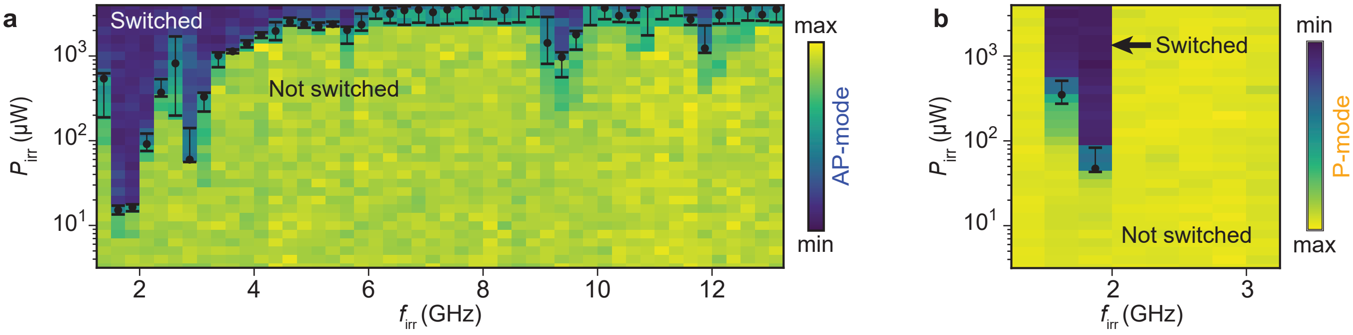

Supplementary Fig. 5. | **Switching-yield maps at** $\mu_0 H_B = 14$ mT **for an independently prepared second sample with nominally identical Py nanostripe arrays.** The YIG film was taken from the same wafer, but, evaporation of the Py film and subsequent process steps were conducted separately from the sample presented in the main text. The yields for magnetic bit writing of this second sample out of a different fabrication batch agree well with the findings presented in Fig. 1 of the main text. The agreement is true for writing magnetic bits, both, under **a** CPW1 and **b** CPW2.

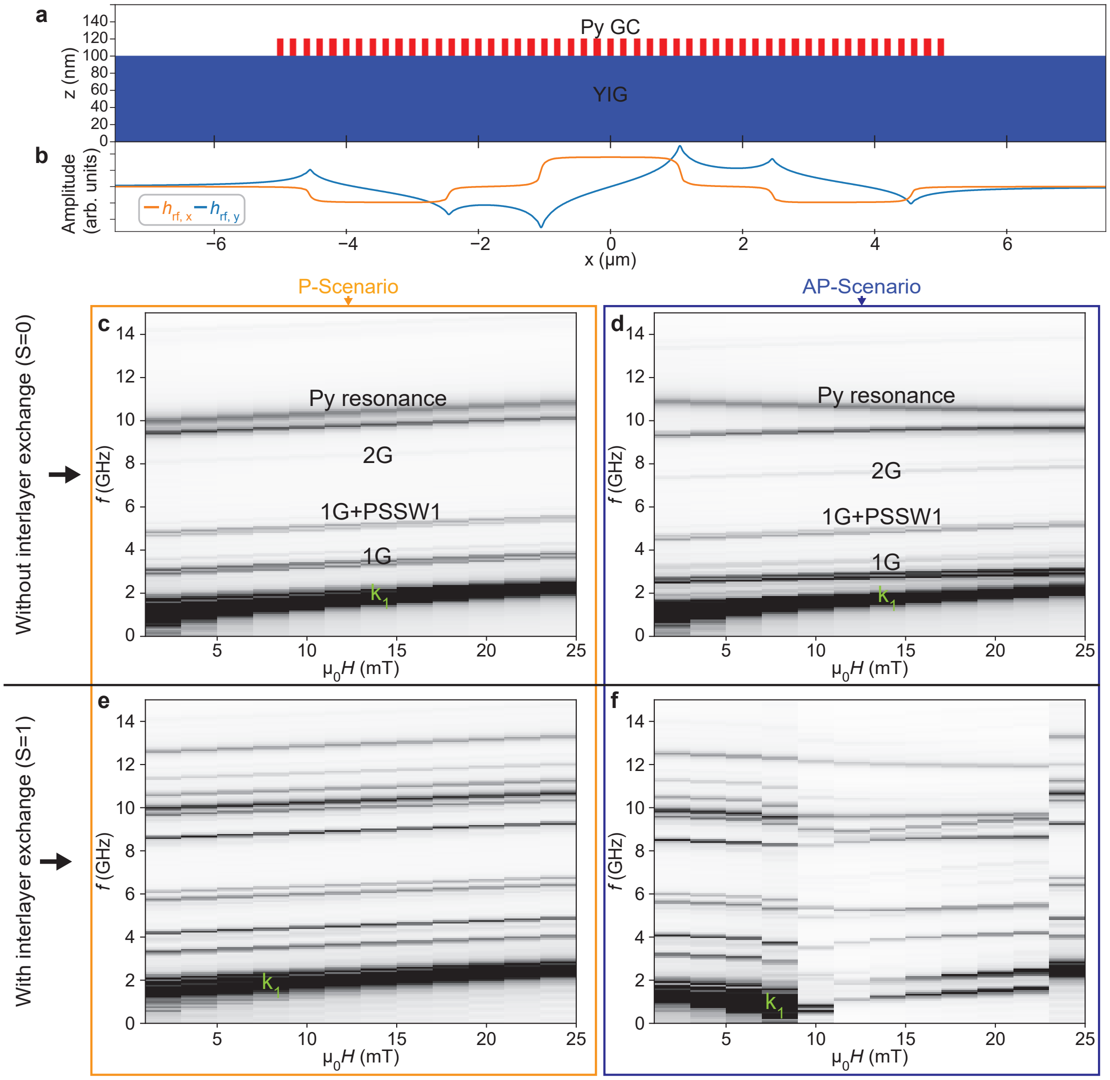

Supplementary Fig. 6. | **Micromagnetic simulations of GC modes in parallel (P)- and antiparallel (AP)- scenario with and without interlayer coupling.** We used Mumax3.10 [1] to simulate the microwave response of the investigated GC sample assuming different interlayer exchange coupling strengths. **a** The simulated geometry consisted of a 100 nm thick YIG film (shown in blue) with a total width of 41 µm. On top of the YIG film was a periodic grating ($a = 200$ nm) consisting of 51 Py stripes (shown in red) with a thickness of 20 nm and width of 100 nm. We simulated a slice of the GC sample in the $x - z$ plane. In y-direction periodic boundary condition was used (with 2048 repetitions in both $+y-$ and $-y-$direction). The grid size was 5 nm × 5 nm × 5 nm. As material parameters we used $\mu_0 M_S = 176$ mT (1005 mT), $A_{ex} = 3.75$ pJ/m (13 pJ/m) and $\alpha = 0.0009$ (0.01) for the YIG film (Py grating). **b** For SW excitation we applied a small $\mathbf{h}_{rf}$. The spatial profile of $\mathbf{h}_{rf}$ was simulated in COMSOL Multiphysics for the nominal CPW dimensions. Following Ref. [2] $\mathbf{h}_{rf}$ was modulated in time by multiplication with a Sinc-function; the SW spectra were subsequently computed from the Fourier amplitudes of the dynamic magnetization components. We simulated the SW spectra for the **(c, e)** P- and **(d, f)** AP-scenario and for the interlayer exchange between YIG and Py either **(c, d)** completely turned off (by setting the scaling parameter S in Mumax3 to zero) or **(e, f)** set to its standard value $S = 1$ for which Mumax3 assumes the harmonic mean of A_{ex} between both layers [1]. In the latter case **(e, f)**, the simulations show a strong modification of the k_1 mode in the AP-scenario, which was not observed in the measured spectra. We thus assume that in the experimentally investigated sample the interlayer exchange coupling was only small or completely absent. When the interlayer exchange was turned off in the simulation, we found a good qualitative agreement between simulation and measured data. In particular, the k_1 mode was not significantly shifted between P- and AP-scenario, while the GC modes (see labels in **(c, d)**) were red-shifted by several hundreds of MHz for the AP-scenario. We attribute this frequency shift in the absence of interlayer-exchange coupling to a modified dynamic demagnetization field. For the simulated GC modes we observed that in the AP-configuration the dynamic out-of-plane magnetization component m_z of the Py stripes was in-phase with m_z of the underlying YIG film, while in the P-configuration there was an π phase shift between them, leading to an increased dynamic demagnetization field.

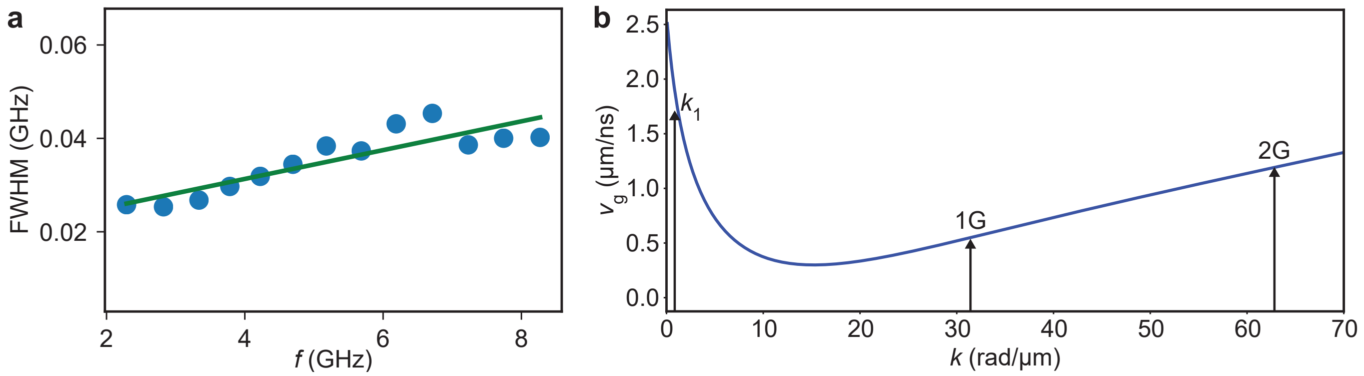

Supplementary Fig. 7. | **Estimation of the SW decay length l_{d} of the k_1 mode at $\mu_0 H_{\mathrm{B}} = 14$ mT. a** To extract the intrinsic Gilbert damping α_{i} of the YIG film, its ferromagnetic resonance (FMR) linewidth was measured with perpendicular-to-plane magnetic fields up to 0.5 T. A CPW with a broad signal line of 20 μm width was used, which excited spin-precessional motion at $k_1' \simeq 0$ rad μm^{-1}. The extracted full width half maximum (FWHM) of the FMR mode in Mag(S11) as a function of its resonance frequency is fitted by a linear function with a slope of $m = 0.0031$ (green line) in **a**. Following Ref. [3] the linewidth broadening depended on α_{i} according to $\delta f = \frac{|\gamma|}{2\pi}\mu_0 \Delta H + 2\alpha_{\mathrm{i}} f$ where ΔH was the inhomogeneous line broadening. δf was the linewidth of the imaginary part, which was estimated by dividing the linewidth of the magnitude Mag(S11) by a factor of $\sqrt{3}$ [4]. Accordingly we calculated $\alpha_{\mathrm{i}} = m/(2\sqrt{3}) = 9 \times 10^{-4}$. **b** Group velocity v_{g} (line) of SWs in a 100 nm thick YIG film as calculated by the Kalinikos-Slavin formalism [5]. v_{g} was calculated for $\mu_0 H = 14$ mT in the Damon-Eshbach configuration ($\mathbf{k} \perp \mathbf{M}_{\mathrm{YIG}}$) using a saturation magnetization $\mu_0 M_{\mathrm{YIG,S}} = 176$ mT, an exchange constant $A_{\mathrm{ex}} = 3.7$ pJ/m and $\gamma = 28.0$ GHz/T as material parameters for the YIG film. At $k_1 = 0.85$ μm/rad (marked by the left black arrow), we extract $v_{\mathrm{g}} = 1.9$ μm/ns. The decay length l_{d} for the SW intensity is calculated by $l_{\mathrm{d}} = \tau \cdot v_{\mathrm{g}}/2$, where $\tau = \frac{1}{2\pi\alpha f}$ is the relaxation time. Using $f = 1.7$ GHz and v_{g} extracted for the k_1 mode, we find $l_{\mathrm{d}} = 99$ μm. The wave vector at 1G (2G) reads $k_{1G} = 2\pi/a = 2\pi/(0.2\ \mu\mathrm{m}) = 31.4$ rad $\mu\mathrm{m}^{-1}$ (62.8 rad $\mu\mathrm{m}^{-1}$)

[1] Vansteenkiste, A. *et al.* The design and verification of MuMax3. *AIP Advances* **4**, 107133 (2014). https://doi.org/10.1063/1.4899186.

[2] Kumar, D. & Adeyeye, A. O. Techniques in micromagnetic simulation and analysis. *J. Phys. D: Appl. Phys.* **50**, 343001 (2017).

[3] Yu, H. *et al.* High propagating velocity of spin waves and temperature dependent damping in a CoFeB thin film. *Appl. Phys. Lett.* **100**, 262412 (2012). https://doi.org/10.1063/1.4731273.

[4] Wang, H. L. *et al.* Scaling of Spin Hall Angle in 3d, 4d, and 5d Metals from $Y_3Fe_5O_{12}$/Metal Spin Pumping. *Phys. Rev. Lett.* **112**, 197201 (2014).

[5] Kalinikos, B. A. & Slavin, A. N. Theory of dipole-exchange spin wave spectrum for ferromagnetic films with mixed exchange boundary conditions. *J. Phys. C: Solid St. Phys.* **19**, 7013–7033 (1986).
